# Supplementary material for: Causal Relationship between Aging and Anorexia Nervosa: A White-Matter-Microstructure-Mediated Mendelian Randomization Analysis
Source: Biomedicines. 2024 Aug 16;12(8):1874. doi: 10.3390/biomedicines12081874 (PMC11351342; doi:10.3390/biomedicines12081874)
Supplement: Supplementary file 1 [file biomedicines-12-01874-s001.zip › Supplementary Figure/Figure S4/Singe SNP plot of MR analysis for TL to ubm-b-1501.pdf]

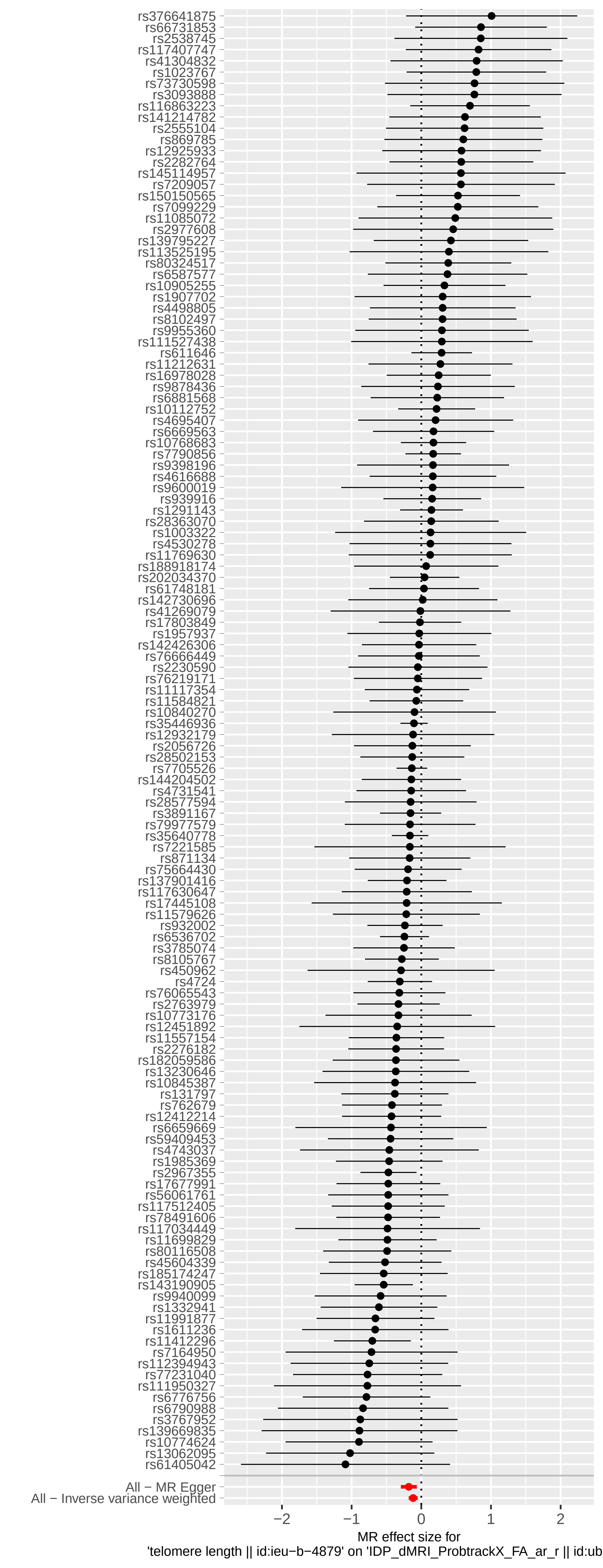
rs376641875
rs66731853
rs2538745
rs117407747
rs41304832
rs1023767
rs73730598
rs3093888
rs116863223
rs141214782
rs2555104
rs869785
rs12925933
rs2282764
rs145114957
rs7209057
rs150150565
rs7099229
rs11085072
rs2977608
rs139795227
rs113525195
rs80324517
rs6587577
rs10905255
rs1907702
rs4498805
rs8102497
rs9955360
rs111527438
rs611646
rs11212631
rs16978028
rs9878436
rs6881568
rs10112752
rs4695407
rs6669563
rs10768683
rs7790856
rs9398196
rs4616688
rs9600019
rs939916
rs1291143
rs28363070
rs1003322
rs4530278
rs11769630
rs188918174
rs202034370
rs61748181
rs142730696
rs41269079
rs17803849
rs1957937
rs142426306
rs76666449
rs2230590
rs76219171
rs11117354
rs11584821
rs10840270
rs35446936
rs12932179
rs2056726
rs28502153
rs7705526
rs144204502
rs4731541
rs28577594
rs3891167
rs79977579
rs35640778
rs7221585
rs871134
rs75664430
rs137901416
rs117630647
rs17445108
rs11579626
rs932002
rs6536702
rs3785074
rs8105767
rs450962
rs4724
rs76065543
rs2763979
rs10773176
rs12451892
rs11557154
rs2276182
rs182059586
rs13230646
rs10845387
rs131797
rs762679
rs12412214
rs6659669
rs59409453
rs4743037
rs1985369
rs2967355
rs17677991
rs56061761
rs117512405
rs78491606
rs117034449
rs11699829
rs80116508
rs45604339
rs185174247
rs143190905
rs9940099
rs1332941
rs11991877
rs1611236
rs11412296
rs7164950
rs112394943
rs77231040
rs111950327
rs6776756
rs6790988
rs3767952
rs139669835
rs10774624
rs13062095
rs61405042
All − MR Egger
All − Inverse variance weighted
−2
−1
0
1
2
MR effect size for
'telomere length || id:ieu−b−4879' on 'IDP_dMRI_ProbtrackX_FA_ar_r || id:ub
